# Interfacial Interactions during Demolding in Nanoimprint Lithography

**DOI:** 10.3390/mi12040349

**Published:** 2021-03-24

**Authors:** Mingjie Li, Yulong Chen, Wenxin Luo, Xing Cheng

**Affiliations:** Shenzhen Key Laboratory for Nanoimprint Technology, Department of Materials Science and Engineering, Southern University of Science and Technology, Shenzhen 518055, China; chenyl@mail.sustech.edu.cn (Y.C.); 11930254@mail.sustech.edu.cn (W.L.)

**Keywords:** nanoimprint, surface modification, demolding force

## Abstract

Nanoimprint lithography (NIL) is a useful technique for the fabrication of nano/micro-structured materials. This article reviews NIL in the field of demolding processes and is divided into four parts. The first part introduces the NIL technologies for pattern replication with polymer resists (e.g., thermal and UV-NIL). The second part reviews the process simulation during resist filling and demolding. The third and fourth parts discuss in detail the difficulties in demolding, particularly interfacial forces between mold (template) and resist, during NIL which limit its capability for practical commercial applications. The origins of large demolding forces (adhesion and friction forces), such as differences in the thermal expansion coefficients (CTEs) between the template and the imprinted resist, or volumetric shrinkage of the UV-curable polymer during curing, are also illustrated accordingly. The plausible solutions for easing interfacial interactions and optimizing demolding procedures, including exploring new resist materials, employing imprint mold surface modifications (e.g., ALD-assisted conformal layer covering imprint mold), and finetuning NIL process conditions, are presented. These approaches effectively reduce the interfacial demolding forces and thus lead to a lower defect rate of pattern transfer. The objective of this review is to provide insights to alleviate difficulties in demolding and to meet the stringent requirements regarding defect control for industrial manufacturing while at the same time maximizing the throughput of the nanoimprint technique.

## 1. Characteristics and Issues in Thermal and UV Nanoimprint Lithography

State-of-the-art functional devices that are related to photonics, electronics, optoelectronics, bioengineering, and information technologies are now commonly fabricated at small scales from various types of materials (e.g., metals, ceramics, polymers, and nanomaterials) [1,2]. Material patterning, which requires lithography techniques to create operative structures, provides unique functionalities and has broad applications in many important engineering fields. Nanoimprint lithography (NIL) is one of the most important nanofabrication techniques among lithographic methods, and it has received widespread attention from both academia and industry in recent years due to its merits of low cost, high throughput, and high feature resolution [3,4,5,6,7]. Particularly due to the daunting technical obstacles and the prohibitive cost-of-ownership of next-generation photolithography systems, NIL has been regarded as a competitive candidate for microelectronic fabrication at 32-nm node and beyond in the international technology roadmap for the semiconductor industry.

Through decades of research, NIL has reached a certain maturity, and commercial NIL systems are available from several vendors for sub-20-nm polymer patterning over substrates larger than 8-inch silicon wafers. However, the implementation of NIL in industrial fabrication is still limited. One of the major hurdles that NIL faces today is the control of the defect density in polymer patterns, because industrial manufacturing, especially the microelectronic lithography step, has very stringent requirements regarding defect control. The NIL process involves mechanically pressing a designed mold into a deformable resist material to create nanostructured patterns [5,8,9]. The resist that is being pressed remains an inverse topography of the template after the imprinting [10]. Because NIL is a molding process, defect generation as a result of the sticking of the polymer to the mold surface is the most difficult to curb in defect management for NIL, which remains a major issue in NIL yield. Though silane anti-sticking coating can be used, defect control is still unsatisfactory for repetitive NIL in volume manufacturing.

Typically, the lithography resolution and fidelity are determined by the pattern size and feature surface roughness of the template [11]. Therefore, the mold fabrication and process parameters are vital to the final NIL resolution and quality. The NIL mold is normally fabricated through photolithography [12], laser direct writer (LDW) [13], or electron beam lithography (EBL) [14,15], and subsequent dry etching and photoresist removal [16,17]. In general, dry etching may cause surface roughness, especially on the pattern sidewalls; increased inductively coupled plasma (ICP) power often increases the etching rate [17]; however, the resultant retarded heat dissipation might create a rough etched surface. Additionally, cycles of etching and passivation are performed by switching SF_6_ and C_4_F_8_ gases in the Bosch process, where scallop structures are usually generated during the etch steps, causing significant roughness on the sidewall [18,19,20,21]. Besides template fabrication, fine-tuning of NIL process parameters is also important in achieving good pattern transfer quality. Artificial neural network algorithms can be implemented on NIL process parameters from experimental and literature data to predict the imprint quality for NIL, which allows users to fabricate high-fidelity structures with less trial and error as well as minimized expense [22].

**Figure 1:**
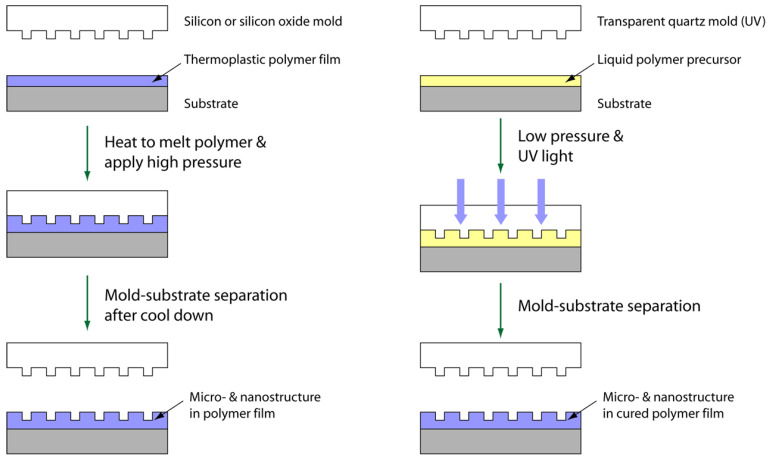
Schematic overview of thermal nanoimprint lithography (NIL) (**left**) and UV-NIL (**right**) processes. The separation between the mold and the micro/nanostructured polymer film in the last step is termed demolding.

Since NIL is mostly a mechanical process, various types of functional materials may be textured to satisfy the needs of different functional devices. Two of the major NIL techniques are thermal NIL and UV-NIL (Figure 1), and both have showed a sub-10-nm resolution [23]. In thermal NIL, the imprinted materials (e.g., a thermoplastic polymer film) must be deformable under applied pressure. In general, the elastic modulus of the resist should be lower than that of the NIL mold during pressing, while the resist maintains adequate reflow behavior to retain the fidelity of the micro/nanostructure. The thermal NIL process is often carried out at an elevated temperature (e.g., above the resist’s glass transition temperature, T_g_) to enhance its fluidity compared to that of the polymer in an ambient environment [9]. The heated resist may flow and completely fill the template cavities under applied high pressure (e.g., a few MPa). The template is then separated after the patterned resist is cooled to a temperature below its T_g_. The dry etching technique, such as ICP, may be performed after imprinting to control the structural height of the micro/nanopattern and remove residual materials [24]. Further, the internal stress relaxation (e.g., stress induced during molding and demolding) may cause reflowing of the imprinted resist [25,26], which is usually undesirable during pattern replication. Recently, we found that the strong chain entanglement in certain polymers seems beneficial to pattern stability and fidelity, and good thermal stability of the nanoscale patterns on polycarbonate is successfully demonstrated at a temperature above its T_g_ [24].

A thermoplastic resist has been proven to be useful for thermal NIL, while a liquid resist that is curable under UV radiation can be utilized in UV-NIL [27]. The UV-curable polymer precursors with low viscosity could flow into the mold cavities and their viscosity could be rapidly increased in seconds via curing under sufficient UV exposure [10]. The fast curing characteristics of the UV-curable precursors seem to make UV-NIL more feasible for high-volume industrial and commercial applications [24]. The UV-NIL process can be carried out at ambient temperature [23] while the mold or the substrate should be UV-transparent. A nanostructured film can be formed through capillary force by pressing the mold onto the multi-dispensed array of low-viscosity UV-curable monomer droplets [23].

**Figure 2:**
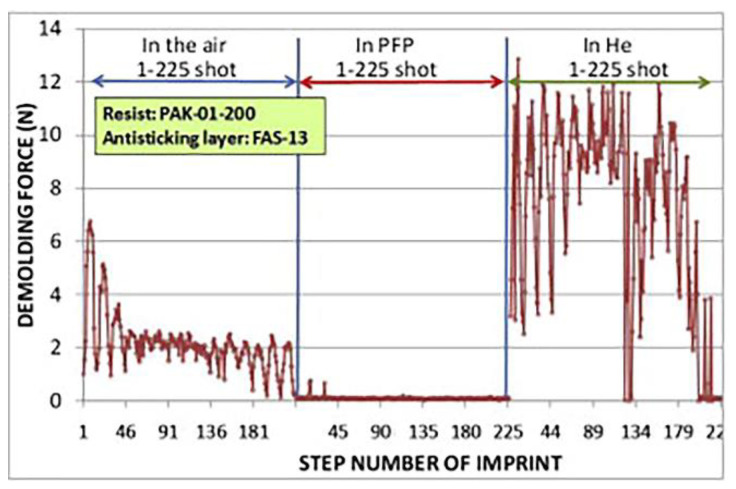
Difference in demolding forces (i.e., maximum force required for separating the mold and the resist after imprinting) between UV-NIL atmospheres of air, pentafluoropropane (PFP), and helium. Reprinted with permission from [34]. Copyright 2015 Elsevier.

A relatively low imprinting pressure may result in the formation of air bubble defects in UV-NIL if the imprinting is performed in air [28,29]. The pinning of the air–liquid interface at the pattern edge, causing incomplete filling of the resist into mold features, is an engineering challenge in UV-NIL [28,30]. Dynamic simulation results predict that the initial fast resist-filling during imprint is pressure-controlled, while the later phase of filling is slower and controlled by the diffusion of entrapped gas [31]. Pinning may take place if there is no pressure gradient between the air–liquid interface and the bulk liquid; hence, there is no driving force to promote liquid flow to fill the mold features [30]. By decreasing the droplet volume to a certain size, the air bubbles can be eliminated because the air volume is small enough to be dissolved into the resist liquid [23,28]. However, this method is not compatible with spin-coated resist films [32]. Conducting the imprinting in a vacuum may be an alternative solution [33]. Imprinting under a helium atmosphere is also reported as a viable solution, which improves resist-filling because the helium gas diffuses faster into the resist compared to that of air due to its small atomic radius [32]. However, neither method can be applied without increasing the manufacturing complexity and costs. Moreover, a helium atmosphere increases the demolding force compared to that of air (Figure 2) [34]. In addition to performing imprinting in a helium environment, the application of a condensable gas (e.g., pentafluoropropane, CHF_2_CH_2_CF_3_; PFP) for bubble elimination was also proposed [34,35]. During imprinting, the volume of the trapped gas decreases and the condensable gas becomes completely liquid [34]. The remaining liquified condensable gas is assumed to remain as a thin PFP-rich layer between the mold and resist, or dissolves in the resist [34,36]. Since PFP contains fluorine and exhibits low surface energy, such a layer is suggested to effectively decrease the release energy (i.e., demolding force; see Figure 2) of the cured resist compared to that in air [34,36,37,38]. On the other hand, the use of condensable gas may be disadvantageous due to pattern height shrinking via absorption by the resist [39]. The resist pattern height was lowered by approximately ~10% as a result of PFP absorbed by acrylate-type monomers [40].

Moreover, the subsequent polymerization of the UV-curable resist may be inhibited by oxygen [41,42,43]. For instance, the oxygen is highly reactive toward the photo-initiated radicals to form stable peroxy radicals during UV exposure, which impedes the following polymer chain growth [42]. The inhibition period due to dissolved oxygen within the resist can reduce the modulus of the cured resist [44], thus extending the required UV exposure time and lowering the process throughput. The diffused oxygen from the ambient environment may also lead to a thin layer of under-cured resist surrounding the perimeter of the mold (detrimental for imprinting); since the inhibition period is a result of photo-initiated radicals reacting with oxygen, altering the photo-initiator concentration and the initiation conditions (e.g., inerting techniques) may help to reduce the impacts of oxygen during the curing process [42].

More recently, some researchers proposed an electrochemical nanoimprint lithography (ECNL) technique by combining NIL and metal-assisted chemical etching (i.e., corrosion at the metal–semiconductor–electrolyte interface), which operated directly on semiconductors such as porous Si [45], n-type crystalline Si [46], and GaAs wafers [47,48]. However, mass transport issues (e.g., blocked mass transfer and local depletion of reactants) need to be resolved before fabricating high-aspect-ratio structures on bulk semiconductors.

## 2. Process Simulations for NIL

### 2.1. Process Simulation for Thermal NIL

In thermal NIL, the process sequence includes hot-pressing and cooling down while the high imprinting pressure is uninterruptedly applied, followed by demolding. During the hot-pressing stage, the temperature is raised above the T_g_ of the resist and the resist is considered to be viscoelastic [49]. The resist flows toward the mold cavities and the maximum compressive stress is concentrated in the vicinity of the outer mold corner [50]. However, fatal defects may not initiate from this step as the resist is mechanically soft above T_g_ and the induced stress may spread to other locations [50]. During the cooling process, stress concentration may result from mold-pressing at a solidified resist (e.g., an elastic body) and the lateral thermal strain induced by the difference in the CTEs between the template and the imprinted polymer [24]. The induced stress is again concentrated at the pattern corner [50], possibly as a result of geometrical constraint or material discontinuities. Template release or demolding is the last but vital process in NIL because adhesion between the resist and the mold may induce destructive pattern replication defects [50].

**Figure 3:**
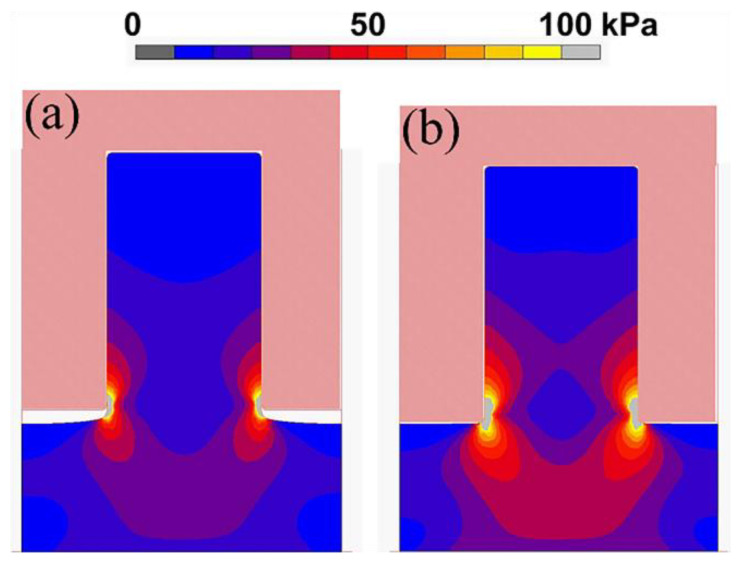
Finite element simulations showing von Mises stress distribution at the moment of demolding. The Young’s modulus values of the polymethylmethacrylate (PMMA) resists are (**a**) 0.5 and (**b**) 5 MPa. The magnitude of the applied detachment force is the same for both resists. Reprinted with permission from [53]. Copyright 2014 American Vacuum Society.

Simulations are useful investigation tools for revealing the physics behind the lithography technique and optimizing the NIL process parameters [49,50,51,52,53,54,55,56]. Computational simulation helps to facilitate a better understanding of the details of the demolding process that may not be easily revealed by experiments (e.g., stress distributions in the resist during demolding), which is essential for optimizing the NIL process. Figure 3 shows the finite element simulation results at the moment of demolding between a silicon mold and polymethylmethacrylate (PMMA) resists with different Young’s modulus values [53]. The modeling result shows that the stress is concentrated at the corner of the pattern [50,55], and the resultant stress field is greater in the resists with a larger Young’s modulus (Figure 3b) [53]. Although the friction force which picks up the resist might not be detrimental, pattern fracture could still occur since the micro-cracks may already have been induced during the previous steps [50].

In addition, complete filling of the polymer resist into the mold cavities during thermal NIL is important for pattern transfer quality. Taylor et al. [57] performed a double-cantilever-beam test to estimate the interfacial fracture work of debonding the mold and the embossed thermal resist, and they found that the demolding work was proportional to an offset of the pattern relief height, which implied incomplete cavity filling, possibly at mold corners. They presented an efficient method to simulate the micro-embossed topography of three thermoplastic resists, namely PMMA, polycarbonate, and Zeonor 1060R (a cyclic olefin polymer), under various processing parameters via a linear viscoelastic model [58]. It was shown that higher embossing temperature resulted in quicker resist penetration into the pattern cavities, while polycarbonate and Zeonor 1060R exhibited higher load sensitivity to cavity penetration (i.e., easier filling) compared to that of PMMA at an embossing temperature of 20 °C above their T_g_ [58]. The accelerated and unified simulation techniques may also predict the locations of pattern replication defects and thus be used as a pre-design check or an iterative tool to guide the chip design process [59]. Further, trapped air within the mold cavities may impede resist-filling. Previous research [60] also demonstrated an extended simulation technique that presumed that trapped air existed and exerted hydrostatic stresses on the mold and resist during embossing. Simulations showed that the trapped air yielded a smaller impact when PMMA was used compared to that of Zeonor 1060R; nevertheless, increasing imprinting load or holding time may allow the escape of remaining air and enable complete resist-filling [60].

### 2.2. Process Simulation for UV NIL

**Figure 4:**
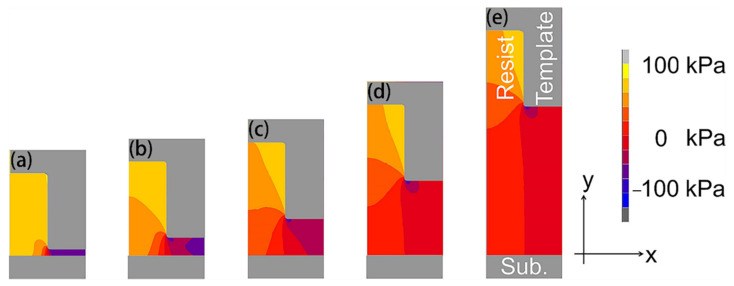
Residual stress σ_yy_ distribution for various residual thicknesses after 0.5% shrinkage, with the resist fully adhering to the template, where the residual thicknesses are (**a**) 10, (**b**) 25, (**c**) 50, (**d**)100, and (**e**) 200 nm. Reprinted with permission from [54]. Copyright 2014 American Vacuum Society.

In UV-NIL, there are several sequential process steps and factors (e.g., resist-filling, optical intensity distribution, and resist profiles after volume shrinkage due to curing) that need to be taken into consideration [52]. In the mold-pressing process, the incompressible resist flows and fills into the mold pattern cavities. During compression, the air may be trapped at the interface between the resist and the mold. Simulation results indicate that the larger features are more quickly filled with the resist compared to that of smaller patterns due to its lower flow resistance, which is thus less likely to dissolute air into the resist [52]. The bubble-trapping issue in small-scale patterns may lead to unfavorable pattern replication defects; however, using a condensable gas may be a plausible solution [34,35]. Additionally, Taylor et al. [61] proposed a theoretical framework to simulate the spreading and coalescence of the dispensed resist droplets underneath the NIL mold, as well as the thickness distribution of the residual layer. They suggested that air entrapment may be alleviated through fine-tuning the template curvatures to accommodate droplet spreading [61]. In the next step, the UV radiation propagates through the transparent mold (e.g., quartz) while the UV-curable resist is polymerized and shrunken. Assuming a small linear shrinkage (e.g., 0.5%) upon curing (i.e., resist still fully adheres to the mold), the volume shrinkage of the resist within the pattern cavity pulls down the mold, which induces a compressive stress in the residual layer (Figure 4). This compressive stress decreases as the residual layer thickness increases, and therefore, the increased residual layer thickness is effective in reducing the demolding load [54].

The optical intensity distribution is correlated with the resist-curing process, where the irradiated beam mostly propagates within the resist region if the pattern line width is equal to the wavelength of the applied UV exposure [52]. The optical intensity in the vicinity of the top portion of the resist may be stronger because of the standing wave reflection from the silicon substrate; thus, the upper portion of the resist yields greater volume shrinkage and residual stress [51,52]. Further, the resultant volume shrinkage of the resist during curing may alter the imprinted feature profiles, and the literature results are somewhat controversial. For example, finite element modeling results indicate a change in the resist pattern height and a subtle variation in sidewall angle [51]. The polymerization primarily reveals a vertical densification, which is somewhat decreasing with increasing pattern aspect ratio (i.e., larger features exhibit more contraction), possibly because the lateral shrinkage strain is fixed at the interface between the cured resist and the rigid silicon substrate [51,62]. In contrast, Tochino et al. reported that the stress induced near the sidewall of the resist is generally larger than that at the upper wall [54]. Nevertheless, both vertical and lateral components of the shrinkage displacements require further investigations due to their impacts on the demolding process. The compressive stress is also large in the residual layer [52,54], which induces squeezing stress toward the pattern sidewalls [63,64,65,66].

### 2.3. Simulations for the Demolding Process

The pattern sidewall roughness may also impact the demolding process. Molecular dynamics simulation results demonstrated that the demolding force increases with the increasing mold sidewall roughness, which is strongly related to the molecular behavior of the resist [53]. For instance, when the resist polymer size is small (e.g., low molecular weights), the resist within the roughness pitch is deformed via easy molecular flow. A further increase in the resist molecular size to a size comparable to the roughness pitch switches the deformation mechanism to molecular stretching. If the resist molecular size is sufficiently large, the resist may not exhibit a large amount of deformation, and thus the demolding force is dependent on the friction force between the template and the cured resin [53].

**Figure 5:**
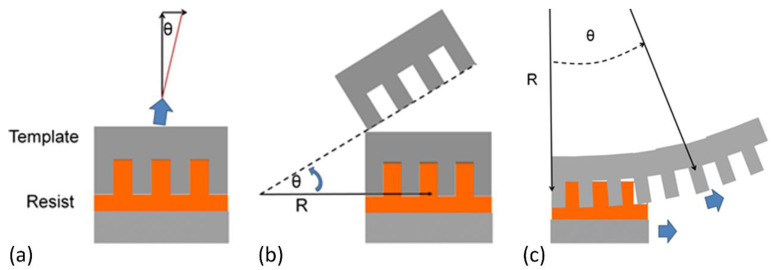
Schematics of demolding process for various release processes: (**a**) lift-off; (**b**) peeling; (**c**) roll-to-roll. Reprinted with permission from [55]. Copyright 2013 American Vacuum Society.

There are various mold-releasing approaches (Figure 5), and the most common method is the vertical lift-off, as discussed previously. However, if the mold is released with an inclined angle with respect to the resist (e.g., peeling and roll-to-roll demolding), a lateral bending stress will be introduced. In the lift-off method, the mold is vertically picked up, though subtle inclination might occur in some real apparatus (Figure 5a). The schematics of peeling and roll-to-roll processes are depicted in Figure 5b,c, respectively. In the peeling process, the simulated maximum local stress decreases with the increasing rotation radius, which may help to attenuate the extent of demolding damage, while in the roll-to-roll process, the maximum induced stress is a function of roller radius, and the resultant stress level appears to be lower compared to that of the lift-off process [55].

## 3. Mold Surface Modifications

### 3.1. Surface Treatment for Nanoimprint Molds

**Figure 6:**
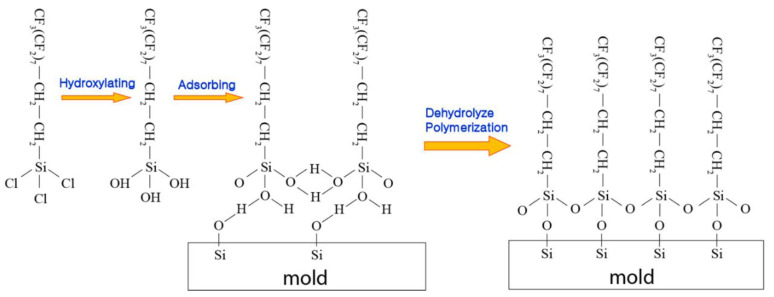
Schematic process showing 1H,1H,2H,2H-perfluorodecyltrichlorosilane (FDTS) monolayer ideally self-assembling on the oxidized silicon surface. By converting the mold surface silanols into inert and hydrophobic –CF_3_ groups with FDTS, adhesion and friction forces can be significantly reduced through bonding site elimination and surface lubrication.

To optimize the NIL process for successful demolding, surface modifications on the template are necessary. The molds (e.g., silicon mold [5,24,67] and metallic mold [68]) can be covered with low surface energy (i.e., a hydrophobic surfactant layer) and low adhesion protective layer for relatively easy demolding. This could not only potentially improve the pattern replication qualities but also prolong the service lifetime of the template through minimizing surface contamination [24,69,70,71]. The protective anti-adhesive films have been extensively studied. For instance, diamond-like carbon (DLC) with low adhesion and reactivity can be deposited on the template using plasma-enhanced chemical vapor deposition, with a mixture gas of methane and argon as the precursor [72,73,74]. The DLC coating exhibits excellent chemical and physical stability during imprinting cycles [72]. It also yields low adhesion energy and effective release performance against methacrylate, vinyl ether [73], and SU-8 resist [74], which might result from its small surface roughness of ~0.2 nm [75]. In UV-NIL, the UV-curable resist is typically cured under UV exposure with a wavelength around 365 or 395 nm, where the UV light may transmit through the patterned template. However, the UV transmittance through DLC is not ideal, and it strongly depends on the film thickness. For example, a 20-nm DLC protective layer only provides ~67% transmittance around a wavelength of 365 nm, while the transmittance declines to ~44% if the film thickness is increased to 45 nm [74]. For comparison, the 365-nm transmittance through quartz is usually ~95%. Moreover, ion-beam-deposited DLC film has relatively poor step coverage with uneven sp^2^ C-C bonding. Consequently, the micro/nano-patterned template with DLC coating may result in spatially non-uniform UV exposure to the underlying resist, causing heterogeneous shrinkage stress distribution and difficult demolding. In addition, protective polytetrafluorethylene (PTFE)-like films can be deposited on the mold through plasma polymerization from a CF_4_/H_2_ microwave discharge, or ion sputtering from CHF_3_ or CF_4_ plasma [76,77]. The first method induces almost 60% of CF_2_ groups among all carbon bonds, while the sputtering technique implants randomly distributed fluorinated ions consisting of mainly CF_3_, CF, and CCF bonds [76]. Both types of PTFE films suffer from degradation during imprinting cycles as a result of fluorine diffusion from the film to the imprinted resist. More recently, a carbon–fluorine polymerized film formed through CHF_3_/O_2_ or C_4_F_8_/O_2_ plasma treatment was demonstrated, where the film was composed of CF, CF_2_, and CF_3_ bonds [78]. The anti-adhesive film created from C_4_F_8_/O_2_ plasma exhibits enhanced mechanical stability and pattern transfer fidelity compared to that of CHF_3_/O_2_ plasma during demolding cycles [78]. Self-assembled monolayer (SAM) films such as tridecafluoro-(1,1,2,2)-tetrahydrooctyl-trichlorosilane (F_13_-TCS) [67], (tridecafluoro-1,1,2,2-tetrahydrooctyl)trichlorosilane (TFS) [79], grafted octadecyltrimethoxysilane (OTMS) [80], and 1H,1H,2H,2H-perfluorodecyltrichlorosilane (FDTS) can also be utilized as a hydrophobic surfactant (Figure 6) [24,70,71].

FDTS may be self-assembled on an oxidized surface through vapor phase deposition [11]. For example, the trifunctional silane heads of the FDTS molecules may interact with -OH groups on a silicon substrate to form Si-O-Si bonds [81]. On the other hand, the hydroxyl group density on the substrate is usually not sufficiently high for every silane to form covalent bonds via dehydrolyze polymerization [82]. Further, the crosslinking among perfluoro-alkylsiloxane molecules might not regularly take place because of steric hindrance [83]. For instance, the size of the fluorine atom is relatively greater compared to that of the hydrogen atoms. The bond length of Si-O-Si is approximately 0.32 nm [83]; on the contrary, the separation distance between Si atoms on the substrate is roughly 0.56 nm [84]. Consequently, robust covalent bonds might only exist at a few of the silicon locations, while the remaining silane molecules are connected to the hydroxide substrate via weaker bonding, such as hydrogen bonding, van der Waals interaction, or highly strained Si–O–Si bonding [85]. The works conducted by Tripp and Hair [86,87,88] also demonstrate that there is no chemical bonding being formed on the surface of substrate; instead, the monolayers might adsorb onto the surface and connect to the hydroxyls on the surface through hydrogen bonding. Hence, the FDTS monolayer may possess a disordered structure, which can reduce the efficacy of the silane coating, and the weakly connected silane molecules might cause SAM film deterioration after a few cycles of the NIL process [89,90].

### 3.2. Stability of Surfactant Coating on Mold

**Figure 7:**
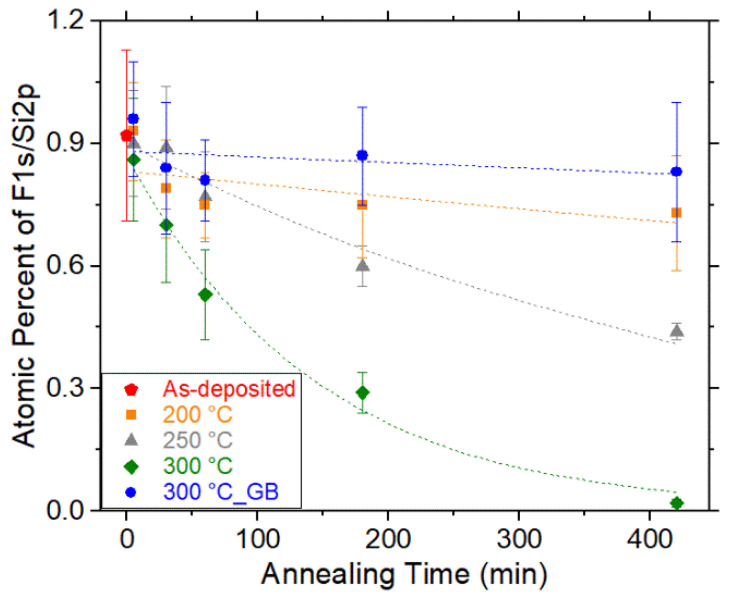
Thermal degradation of the FDTS monolayer displayed by the change in fluorine coverage (i.e., F 1s/Si 2p peak areas normalized by sensitivity factors) as a function of annealing time and temperature. The glove box is abbreviated as GB. Reprinted with permission from [11]. Copyright 2020 American Vacuum Society.

Since heating (around ~100–250 °C) is usually employed in thermal NIL, there are a few works which have investigated the thermal stability of the monolayers [81,85]. They showed that annealing at 100–200 °C can lead to considerable fluorine loss [85], and the water contact angle decreases dramatically when SAM is annealed at 400 °C for 2 min [81]. More recently, we measured fluorine coverage (i.e., F 1s/Si 2p total peak area ratio normalized by the sensitivity factor of each element) loss at various annealing times and temperatures using X-ray photoelectron spectroscopy (XPS) [11]. Generally, fluorine coverage subsides as the annealing time is increased (Figure 7), possibly due to chain scission and/or desorption of the monolayer. The observed change in the desorption rate may result from the monolayer packing details and the intermolecular heat transfer [91,92,93]. The evolution of the fluorine coverage and the contact angle indicates that the anticipated useful service lifetime of the monolayer is ~180 min upon annealing at 300 °C and ∼560 min if annealed at 250 °C [11]. In addition, aggregations were observed on specimens annealed in an ambient environment [11], possibly because of the existence of vacant -OH groups and moisture molecules in air [11]. The formed aggregations notably deteriorate the thermal stability of the coatings [81,94]. In contrast, almost no chain scission or desorption was observed for specimens heated in an inert environment with minimization of reactive oxygen and water molecules (i.e., glove box, H_2_O ≤ 0.1 ppm; O_2_ ∼ 5.9 ppm) [11]. The experimental thermal degradation activities in air and inert environments offer a valuable strategy for designing a pragmatic NIL process for the commercial production of micro/nanostructured polymeric materials.

**Figure 8:**
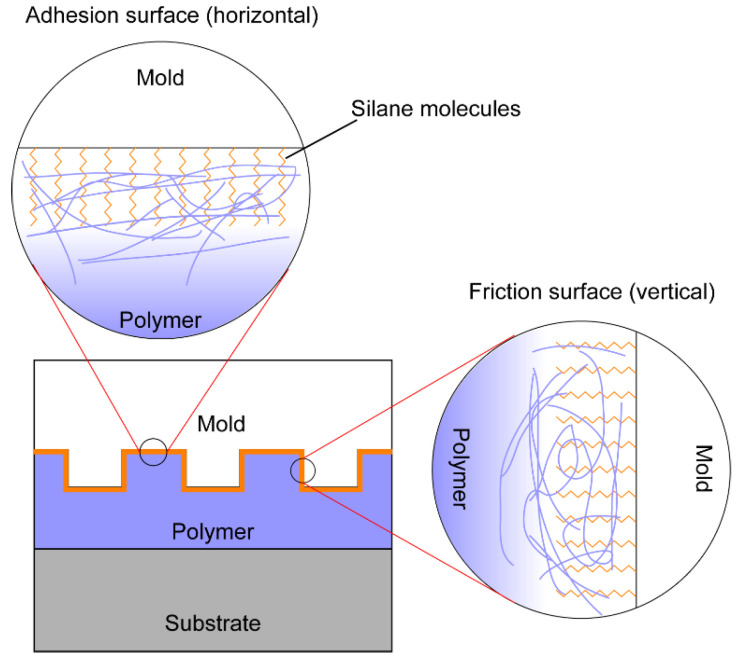
Schematic of chain entanglement between silane molecule and polymer resist.

In UV-NIL, the silane monolayer will be in contact with the liquid polymer precursor and exposed to UV light. Polymer resist can partition into the silane monolayer by occupying the free volume in the silane monolayer (Figure 8). The wetting of the polymer liquid on the silane-coated mold surface also has a significant impact on chain entanglement. A small contact angle usually means higher phase compatibility or mutual solubility across the interface, which usually leads to a higher degree of chain entanglement. Chain entanglement between the silane molecules and the polymer chains in NIL will have a significant effect on the adhesion and friction properties between the mold surface and the polymer film, which is still largely unexplored in NIL research. Silane stability will also be affected due to chain scission by UV light [95] or by increased adhesion and friction forces from chain entanglement. In addition, the SAMs may interact with the free radicals in the crosslinked resists [96], which causes a rise in the surface energy of the template [97].

It has been demonstrated that the FDTS monolayers suffer from permanent wearing after only a few cycles of the demolding process, with substantial loss of fluorine atoms, where the surface roughness increases remarkably [73,74]. The wearing of the silane monolayer will gradually increase both the adhesion and the friction stresses, because the silane wearing will change the surface properties of the template and the chemical composition of the polymer surfaces (e.g., broken silane chain embedded in polymer phase surface). The erosion of the silane monolayer on a mold surface can deteriorate its functionality as an anti-sticking layer and lead to progressively higher defect density after many cycles of imprinting. Silane recoating becomes imperative at the point when the defect density of the patterned polymer becomes unacceptable. This required silane re-processing will inevitably slow down the production rate. Since NIL is being commercially utilized for the production of polymeric optical devices [11], an assessment of the useful service lifetime of the anti-sticking protective film through monitoring of the physical and chemical changes as wearing progresses must be carried out to determine the optimal time interval between two successive silane coatings.

Due to intermolecular vdW interactions, long-chain silanes have been shown to form a more ordered monolayer film. Higher surface coverage and chain ordering can be achieved compared with short-chain silanes under the same processing condition; thus, ordered long-chain silanes are more effective in adhesion and friction reduction for inorganic surfaces. However, in both thermal and UV-curing NIL, the silanes are in direct contact with the polymer resist. Heating and high pressure used in thermal NIL will inevitably cause chain entanglement between the silane molecules and the polymer matrix. In UV-NIL, precursor liquid can impart into the free volume in the silane monolayer and lock silane chains in the cured polymer matrix after UV curing. In both cases, the chain entanglement can contribute to a large separation force required to split the mold and the substrate after imprinting. An entangled interphase as thin as 1 to 2 nm is sufficient to increase the adhesion by an order-of-magnitude, and the chain lengths of OTMS and FDTS are in the order of 2 nm [98]. Thus, the adhesion and friction forces can be significantly reduced by using silanes of shorter chains. However, there is a potential problem with short-chain silanes. Due to the higher molecular mobility and weaker interchain vdW interaction, it is difficult to obtain a monolayer with highly ordered packing and high surface coverage. This problem could potentially be solved by lowering the temperature at which the monolayer is prepared [99].

### 3.3. Surfactant Additive in Resist as Inner Release Agent

**Figure 9:**
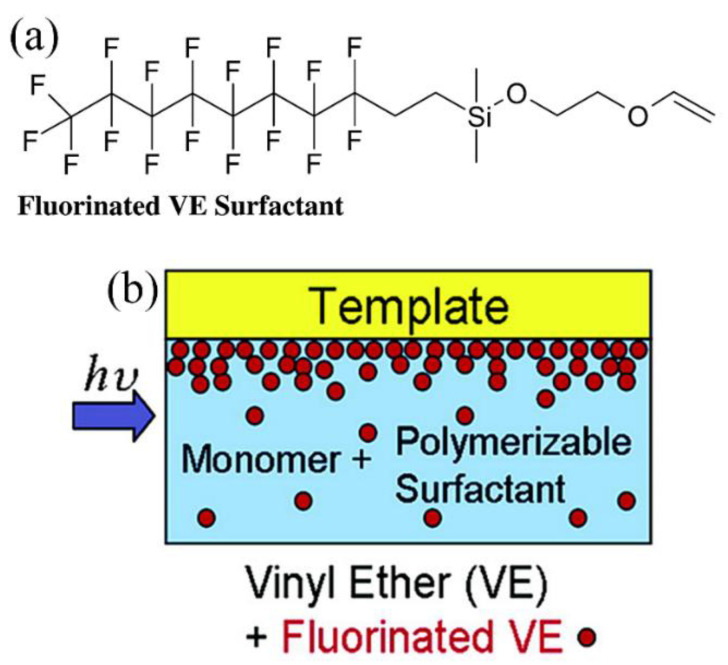
(**a**) Structure of the vinyl ether (VE) monomers; (**b**) segregation of the surfactant molecules to the polymer resist–mold interface. Reprinted with permission from [100]. Copyright 2007 American Chemical Society.

Furthermore, inner release agents such as fluorinated surfactants (e.g., fluorinated vinyl ether; Figure 9a) can be added into the polymer resist [34,100]. Experimental results indicate that the average demolding force significantly decreases and the anti-sticking layer degradation is deferred with the surfactant added to the resist compared to that of the resist without the inner release surfactant [34]. These benefits of the inner release agents can be attributed to the segregation of the surfactant molecules to the polymer resist–mold interface (Figure 9b) [100]. Previous XPS and surface tension investigations found substantial segregation of fluorinated surfactants to the interface between polymer and air [101,102], fluorinated SAM-treated quartz [100], and anti-sticking-layer-coated silicon [34], respectively. In contrast, negligible segregation was detected to the interface between polymer and glass [101], quartz [100], and silicon [34], respectively. The driving force for the fluorinated surfactant segregation was proposed to be related to the low polarity of the air and the low surface energy of the template [100]. Hence, a fully dense SAM might not always be necessary if the inner release agents were added within the monomer [100]. As long as the surface energy remains sufficiently low, the surfactant segregation on the polymer–mold interface may occur and mold durability may be preserved [100].

### 3.4. Surface Modification by ALD

The mold can also be coated with a protective layer through atomic layer deposition (ALD), which is a vapor-phase deposition method for depositing ultra-thin and conformal coatings with atomic-level precision control of film growth [103,104,105]. ALD may be utilized to reinforce the template durability, and the polymeric photoresist can be directly used as an imprinting template after it is conformally coated with ALD film [104]. Additionally, ALD offers new insights to fine-tune the surface structures. For instance, ALD was used to precisely and accurately alter the ridge width of metallic gratings [103] and the pattern size of hole/pillar arrays [103,104,105,106]. The refining of pattern resolution from 180 to 85 nm was reported via depositing an alumina layer through ALD [104]. Therefore, the conformal and uniform film deposited through ALD may be used to modify the surface morphology, for instance, on the pattern sidewalls.

In addition, resist adhesion to the mold surface sometimes can cause “pulling out” of defects in the transferred resist pattern, since the patterned mold usually has a larger contact surface area. Mold surface modifications decrease the release energy and reduce such defects. In the meantime, forming an adhesion layer to increase the adhesion between the substrate and the cured resist (i.e., suppress de-wetting of the resist layer) may also be considered [34,107].

## 4. Interaction Forces between Mold and Resist during Demolding

### 4.1. Origins of Demolding Forces in Nanoimprint

**Figure 10:**
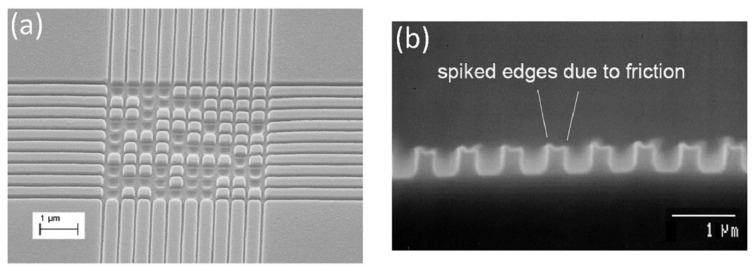
Polymer pattern defects after NIL. (**a**) Polymer pattern missing due to polymer sticking on mold; (**b**) spiked edges in polymer pattern due to friction forces.

A major issue in the NIL process is the high defect rate as a result of mechanical pressing, which causes difficult demolding [50,54,108], as also briefly discussed in the previous sections. The adhesion and the lateral and friction forces [109] that originate during imprinting cause the fracture of the mold and/or the resist [50], as well as strong adhesion between the mold and the resist [89,90]. For instance, due to adhesion and friction, the molded polymer film tends to stick to the mold surface, causing a missing pattern in the polymer film (Figure 10a) or polymer pattern deformation after NIL (Figure 10b). For successful patterning of a polymer film on a substrate, the forces acting on the mold–polymer interface must be minimized. The contact adhesion that comes from the attractive van der Waals (vdW) force is usually a small component of the overall adhesion. Bonding across the interface, including chemical bonding and hydrogen bonding, can contribute significantly to the adhesion and friction forces. This is especially true for silicon and silicon oxide (the most commonly used mold materials) surfaces, where many dangling hydroxyl groups can readily form chemical and hydrogen bonds with polymer chains, which leads to a very large adhesion force. The lateral and friction forces are usually attributed to the thermal stress, as a result of the CTE difference between the template and the resist [24], or volume shrinkage of the UV-curable precursor upon exposure [110].

### 4.2. Thermal Stress in Nanoimprint and the Impact of Demolding Temperature

In the thermal NIL process, internal stress could build up in the polymer, which may result in unwanted structure-transferring defects or pattern cracking [111]. The internal stress relaxation [111,112] or shape recovery [113] after imprinting may also impact the long-term stability of the structured polymer. The previous literature proposed that internal stress accumulation consists of two components. First, when a polymer is heated above its T_g_, the forced flowing of the polymer melt into the cavities under volume conservation accumulates internal stresses [49,111,114]. However, these internal stresses may be relaxed or spread over the polymer as a result of polymer molecule disentanglement while the polymer is at its viscous regime above its T_g_ [50,111,114]. Second, during the cool-down step, a mold-imprinting pressure below T_g_ may induce stress concentration at the corner of the pattern [50]. Additionally, the difference in CTEs between the mold and the resist may build up thermal stress and usually lead to a compressive pressure against the polymer [24,111]. The lateral thermal stress can be significant as a processing temperature of a few hundred degrees (°C) may be required during imprinting. The resultant friction force could lead to pattern fracture during demolding as the critical defects might already be induced from the previous cool-down step [50]. Experimental investigations indicate that a slow cooling process improves PMMA pattern transfer quality [50]. The slow cooling rate may introduce a lower thermal strain rate, thus affecting the fracture strain of the material (i.e., inhibiting embrittlement) [115,116]. Besides pattern cracking, residual deformations and residual stress relaxation may impede post-processing of the imprinted material [112]. A systematic experimental investigation [111] demonstrated that residual deformations essentially have no dependence on the imprinting temperature, time, and pressure; instead, they are dependent on the cooling conditions (e.g., demolding temperature). For instance, the imprinted PMMA that was demolded at 100 °C shows nearly no shape change after demolding and post-baking (i.e., to accelerate the relaxation processes), as a result of minimal residual stress [111]. In contrast, the samples demolded at 70 and 40 °C exhibit anisotropic shape recovery, which can be catastrophic to pattern post-processing [111]. From the perspective of demolding force, an optimal demolding temperature was determined to be approximately ~85 °C for a PMMA resist [111].

### 4.3. Resist Shrinkage in UV-NIL and Its Impact on Adhesion and Friction Forces

On the other hand, the chemical bonds between resist molecules switch from van der Waals to covalent during UV-induced curing, and macroscopic densification has been reported in the UV-NIL process [10,62]. The lateral resist shrinkage upon curing is a dual influence between the volume shrinkage of the residual layer (i.e., resist not filled into patterned mold cavities) pushing the resist against the pattern sidewalls [63,64,65,66] and the shrinkage of the polymer confined within the mold cavities that locally compact themselves [109,117]. Usually, the shrinkage force induced by the residual layer is greater; thus, the combined shrinkage force may push the resist within the cavities against the vertical pattern sidewalls [109]. Hence, a thin and uniform residual layer is highly desirable for UV-NIL [34]. The frictional force acting on the sidewalls, which was initiated from the lateral compressive stress, may be substantial for some features with high aspect ratio and rough sidewall surface. Therefore, the demolding process frequently causes pattern replication errors and defects. The demolding process requires further investigations in order to optimize UV-NIL parameters with improved demolding characteristics [10].

The adhesion and friction forces can be measured both in microscale and in macroscale. In the literature, atomic force microscope (AFM) nano-indentation tests were utilized to measure the adhesion force required to separate the AFM tip and the indented resist [90]. Adhesion force can be obtained from the pullout characteristic of the cantilever beam on a polymer surface, while the friction forces can be measured by the lateral deflection of the cantilever beam in AFM when the tip slides on a polymer surface. However, this approach only focuses on a small region, whereas the contact area in nanoimprint is usually at wafer scale [118]. Macroscale force measurement directly quantifies the minimum separation force required to split the mold and the substrate after imprinting. Some researchers [10,118,119,120] have demonstrated a more practical method to measure the demolding force through a uniaxial tensile system, which simulates the parallel demolding procedure. Other experimental research demonstrated the relationship of the total demolding force versus the volume shrinkage of the UV-curable resists [10,51,109,110]. In order to minimize the shrinkage upon curing, approaches such as pre-exposure to induce a pre-cured residual layer before imprint [121], stepwise polymerization [122], pulse UV curing [123], addition of inorganic nanoparticles [124], reducing the concentration of the photocurable components [125], introducing disulfide bonds [126], using monomers with bulky pendant groups [62], or adding the volume-expanding monomer [110] in the polymer formulation have been reported.

**Figure 11:**
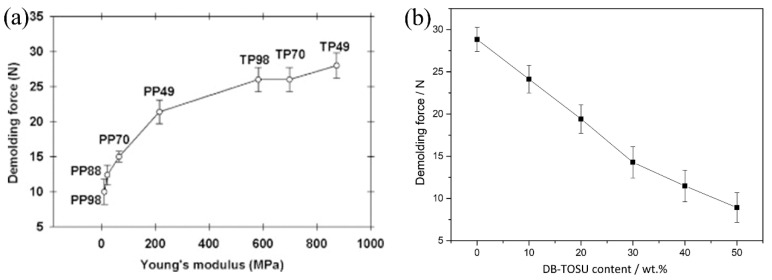
(**a**) Demolding force dependence on resist’s Young’s modulus and crosslinking agent content for polypropyleneglycol diacrylate (PP) and tripropyleneglycol diacrylate (TP). The coding digits show the amount of the base (TP or PP) in weight percent, e.g., PP98 represents 98 wt% base PP, 2 wt% photoinitiator (kept constant), and 0 wt% crosslinking agent. Reprinted with permission from [120]. Copyright 2011 Elsevier. (**b**) Demolding force as a function of volume-expanding monomer 3, 9-Diethyl-3, 9-bis (allyloxymethyl)-1, 5, 7, 11-tetraoxastetraoxaspiro undecane (DB-TOSU) content. Reprinted with permission from [110]. Copyright 2019 Elsevier.

Amirsadeghi et al. [120] demonstrated that the elastic modulus of the resist can be reduced through modulating the resist’s crosslinking agent content, which led to decreased adhesion at the resist–mold interface, thus facilitating demolding. When a small amount of crosslinking agent is present, the volume shrinkage and the resultant shrinkage stress during curing are also attenuated [127]; therefore, demolding force may not lead to the fracture of patterned structures. However, the influence of the crosslinking agent is only pronounced in resists with a longer oligomer length. This is because resists with a shorter oligomer length exhibit a T_g_ higher than the ambient temperature and thus a relatively larger elastic modulus even without any cross-linking agent (Figure 11a) [120]. In addition, Min et al. [110] formulated a novel nanoimprint resist by copolymerization of epoxy and a monomer that undergoes volume expansion due to double-ring structure expansion during acid-catalyzed polymerization. They showed that both the volume shrinkage of the synthesized resist and the consequent demolding force can be diminished by increasing the volume-expanding monomer content (Figure 11b) [110]. In the meantime, the mechanical strength of the cured resist dramatically deteriorated, which may eventually undermine the subsequent patterning due to severe pattern relaxation after imprinting. Further optimization through introducing additional additives into the resist formulation is needed. In addition, some researchers proposed the most appropriate resist mold candidate through measuring the total adhesion force between different molds and commercially available photocurable resists. For example, Perumal et al. [118] compared various template–commercial resist combinations via demolding force measurement, and they found that a high-molecular-weight perfluoropolyether dimethacrylate (H.M.PFPE) template yielded the best release property when AMONIL MMS10 (AMO GmbH, Germany) was utilized as the resist. Taniguchi et al. [119] found that a PAK01 (Toyo Gosei, Japan) UV-curable resist showed smaller adhesive stress compared to that of TSR820 (Teijin Seiki, Japan) when slide-glass was used as a mold.

**Figure 12:**
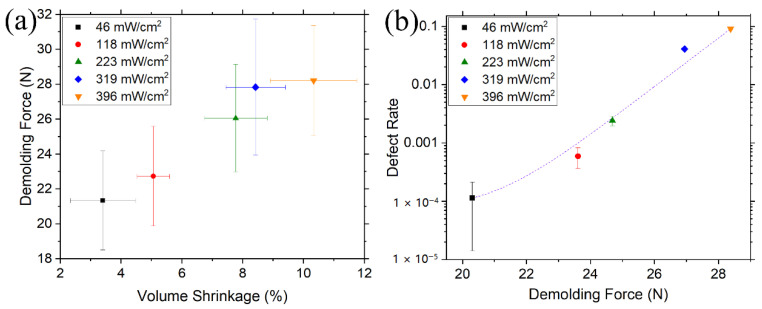
(**a**) Correlation between polymerization volume shrinkage and measured demolding force of the UV-curable resist when exposed to 365-nm UV radiation at various intensities for 15 s. (**b**) The correlation between the demolding force and the resultant defect rate for the resists cured under different UV exposures. Reprinted with permission from [10]. Copyright 2020 IOP Publishing, Ltd.

Recently, we reported that the volume shrinkage could be effectively lowered to ~3% through minimizing applied UV exposure [10]. The trend of decreasing demolding force with the decreasing volume shrinkage is clear (Figure 12a). These results were attributed to the combined influences of the modification of the adhesion and the friction forces as a result of adjusted degree of crosslinking of the polymer resist. Chan et al. showed that a softer template can improve the demolding process [128]. A resist with a lower elastic modulus (resulting from a lower degree of crosslinking) may exhibit decreased adhesion force [129], while reduced volume shrinkage decreases the normal force against the structure sidewalls. These two effects in combination lead to a demolding force reduction. Further, the quantitative experimental investigation shows that the pattern transfer defect rate yields an exponentially rising tendency as a function of the demolding force (Figure 12b). For a resist cured under a slight UV intensity, the majority of the induced stress during demolding is stabilized by plastic deformation of the softer resist while suppressing pattern cracking; in contrast, the resists cured under a large amount of UV irradiation with higher strength may dissipate only a small amount of heat [10]. The brittle failures of either the template or cured resist may take place due to the sudden release of unaccommodated strain energy. Therefore, the degree of crosslinking and the consequent volume shrinkage of the polymer resist upon exposure must be carefully optimized before separation from the mold [10].

The experimental results indicate that the diminished volume shrinkage of the cured polymer is typically associated with the deterioration of its mechanical strength [110]. Even though a mechanically softer resist seems beneficial for the demolding procedure [120], a soft resist might not endure the pulling during demolding, where pattern relaxation takes place [110]. Hence, as long as critical pattern relaxation is not detected, the minimal amount of UV intensity would lead to a smaller total separating force, which considerably reduces the risk of creating defects [10].

### 4.4. Decoupling Adhesion and Friction Forces during Demolding

Though adhesion and friction forces contribute differently to the polymer-sticking on the mold, as shown in Figure 10, most of the current studies in NIL do not distinguish them, and the sum of the two forces is regarded as the total “adhesion” force [130]. Since actual adhesion and friction forces come from horizontal and vertical surfaces on the mold, respectively, their relative sizes in the overall mold–polymer holding force are determined by the aspect ratio of the mold pattern. A higher aspect ratio is always desired for easier processing after NIL, but the increased vertical surface area inevitably increases the undesired friction force. Knowing the relative sizes of the adhesion and the friction forces allows us to optimize the mold depth. In patterning very high-aspect-ratio polymer structures, friction force may dominate and lead to low yield, which presents a great challenge in creating very high-aspect-ratio polymer structures for specific applications such as polymeric microneedles for drug delivery. Although metal, glass, silicon, and ceramics are also fabricated as microneedles, the rigid piece may break inside the skin, causing pain and possibly swelling of the skin [131]. On the contrary, polymers are preferred, because they absorb water into the polymeric network, leading to swelling of the microneedle and intact removal from the skin [132]. Additionally, due to surfactant chain entanglement, the pullout of the silane chain from the polymer phase contributes differently to the adhesion and the friction forces. For adhesion, the silane chain is pulled out perpendicular to the mold–polymer interface during separation, while for friction, the silane chain is pulled out with shear movement. It is thus necessary to evaluate adhesion and friction forces separately for various types of silane coating in order to make an intelligent choice of silane chain length for specific mold pattern.

**Figure 13:**
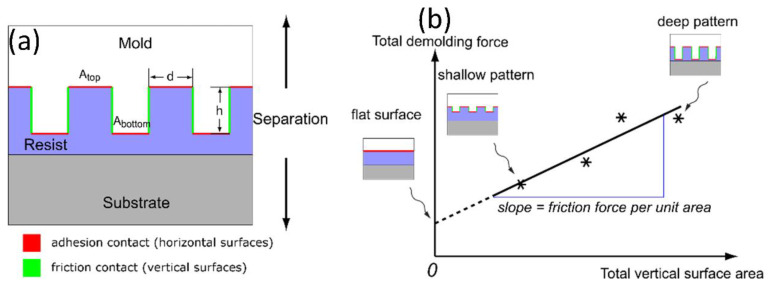
(**a**) A schematic of the adhesion and friction surfaces and pattern geometries; (**b**) a schematic of data acquisition and interpretation to extract adhesion and friction forces in NIL. Reprinted with permission from [133]. Copyright 2021 Elsevier.

Since adhesion and friction forces affect the polymer pattern in different manners, understanding the distinction between them is important for providing strategies of designing the pattern feature and thus minimizing the total separation force as well as pattern replication defects during the demolding process. Amirsadeghi et al. [109] developed a new methodology to enable direct measurement of the adhesion and the friction stresses individually. A schematic of this novel measurement technique is shown in Figure 13. First, molds with equivalent patterns but various aspect ratios are fabricated. The molds are then used to imprint into a polymer layer on a substrate by UV-NIL. Because of the different mold aspect ratios, the mold–polymer contact interface increases as the aspect ratio increases. The total surface responsible for adhesion (red horizontal lines in Figure 13a) remains almost constant; on the other hand, the total friction surface (green vertical lines in Figure 13a) amplifies for molds with higher aspect ratios. The total separation force can then be obtained using a macroscale force measurement tool. By measuring a series of total demolding forces versus mold structural height, where the adhesion surface area is fixed while the friction area increases with the pattern depth, the adhesion and the friction stresses can be extrapolated. As shown in Figure 13b, the extracted slope represents the friction stress, while the line interception gives the adhesion stress. These measured forces can then be used to estimate the actual holding forces between the mold and the polymer resist.

**Figure 14:**
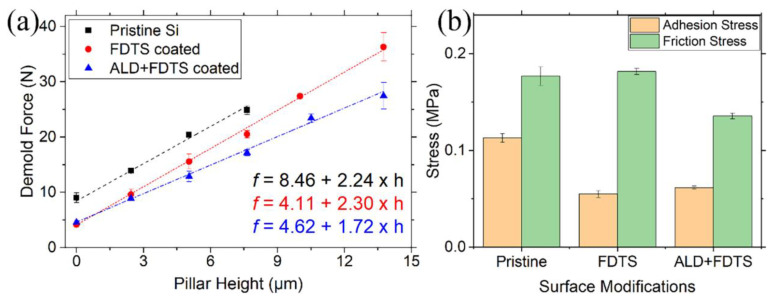
(**a**) Demolding force versus structural height of a pillar array mold with various surface modifications. (**b**) The fitted slope and intercept were used to calculate adhesion and friction force per unit area. Reprinted with permission from [133]. Copyright 2021 Elsevier.

Amirsadeghi et al. [93] measured demolding force as a function of grating pattern height for resists with various compositions. The demolding force increased with the stamp depth, and the obtained shrinkage stress was typically greater than the adhesion stress. They also measured the shrinkage stress for pillar structures, where the resultant shrinkage stress was larger compared to that of gratings with similar linewidth. It was plausible that the resist shrunk in all directions against the pillars, while the resist may shrink without any constraint in the direction parallel to the gratings [93]. We measured the demolding force (Figure 14a) between the ALD-covered pillar molds and the cured resists in UV-NIL, and the corresponding results were compared to the molds with low-energy surfactant coatings for easy mold release and pristine silicon molds, respectively [133]. Since the geometry of the template was known, the adhesion and friction stresses (Figure 14b) were extrapolated from the zero intercept and the slope of the linear fit described in Figure 13b for each surface modification. The silicon pillars coated with FDTS yield attenuated adhesion stress while friction stress remains unchanged compared to that of pristine silicon mold because the friction coefficient of the rough sidewall might be the predominant factor controlling the magnitude of the friction stress. Further, the silicon molds covered with an ALD-induced conformal layer plus FDTS monolayer exhibit lowered friction stress, though their adhesion stress is comparable to that of samples covered with only FDTS. Though the friction stress diminishes by only 25.3% compared to that of the template with merely an FDTS coating (Figure 14b), a slight decrease in the demolding force may lead to a great improvement in the transfer quality. In general, the extrapolated friction stress acting on the sidewalls is greater than the adhesion stress on all specimens. Therefore, approaches to reduce both adhesion and friction stresses are necessary, especially for high-aspect-ratio patterns where the friction force can be large enough to disrupt the polymer pattern during demolding. On the other hand, a shallow mold structure may reduce the friction stress and exhibit a lower defect density. In view of our experimental results, ALD along with FDTS coverings on the imprint mold effectively decrease the total demolding force. In addition, the sequential multi-layer film (e.g., ALD and FDTS layers) demonstrated improved mechanical and chemical stabilities of the hydrophobic monolayer [134]. The improved stabilities were attributed to the augmented density of the reactive hydroxyl groups on the ALD-grown oxide layer for covalently binding the fluorinated silanes [134], consequently resulting in high coating quality of the monolayer.

## 5. Conclusions

Nano/microstructured materials with novel or enhanced properties have many useful applications in modern devices. NIL is a viable method for pattern replication on various materials, and it carries both low cost and high throughput compared to conventional lithography approaches such as EBL and photolithography. Both thermal and UV-NIL impart difficult separation between the template and the imprinted polymer during demolding, as a result of substantial compressive strain accumulation induced by CTE mismatch or volume shrinkage upon curing. The factors contributing to defect generation in NIL and possible solutions to alleviate difficult demolding, such as alternating resist materials, employing imprint mold surface modifications, and finely controlling NIL process parameters, are deliberated. Overall, this review on interfacial interaction between the mold and the resist aims to bring insights into optimizing the NIL process and to inspire new approaches to minimize interfacial interactions to mitigate defect generation during demolding. Although there is already much research on the nanoimprint process, further investigations into defect mechanisms are still needed to fully exploit the potential of NIL in commercial manufacturing of high-resolution structures for electronic, photonic, and bioengineering applications.
